# Virus-directed CAR immunotherapies for chronic HBV and HIV: a systematic synthesis of preclinical and early clinical evidences

**DOI:** 10.3389/fmed.2026.1768365

**Published:** 2026-03-26

**Authors:** Sirwan Sleman, Masood B. Ameen, Omed I. Abid, Barham J. Abdullah, Zaniar A. Abass

**Affiliations:** 1 College of Veterinary Medicine, University of Sulaimani, Sulaymaniyah, Iraq; 2 Nursing Department, National Institute of Technology, Sulaymaniyah, Iraq; 3 Sulaimani Private Veterinary Hospital, Sulaymaniyah, Iraq; 4 Masi Altuni Company, Sulaymaniyah, Iraq

**Keywords:** CAR immunotherapies, chronic HBV, chronic HIV, immunotherapy, virus-associated CAR therapies

## Abstract

**Background:**

Chronic hepatitis B virus (HBV) and human immunodeficiency virus (HIV) infections are among the most important global health issues. Virus-directed CAR-T and CAR-NK are promising strategies capable of targeting virally infected cells. The therapeutic potential, safety, and translational readiness of these platforms have not been fully synthesised.

**Objectives:**

This study assessed preclinical and early clinical evidence of CAR-T and CAR-NK immunotherapies against HBV and HIV, including efficacy, safety and translational feasibility.

**Methods:**

Databases were searched according to PRISMA 2020 guidelines. For this review, eligible studies included *in vitro*, *in vivo*, and clinical studies examining virus-directed CAR lymphocytes. Random-effects models generated pooled standardised mean differences (SMD) and risk ratios (RR). The level of specific evidence was considered by GRADE.

**Results:**

Forty-three studies met the inclusion criteria (21 *in vitro*, 14 *in vivo*, and 8 clinical). Preclinical HIV CAR-T models demonstrated significant reductions in HIV p24 antigen levels (pooled SMD = −1.15, 95% CI −1.50 to −0.80). Similarly, HBV-directed engineered T-cell studies showed a marked decrease in HBsAg and HBV DNA (SMD = −1.30, 95% CI −1.70 to −0.90). CAR-NK platforms displayed comparable antiviral activity with potentially improved safety profiles. In vivo analyses also indicated consistent suppression of HIV RNA (SMD = −0.92, 95% CI −1.26 to −0.58) and moderate reductions in HBV DNA levels (SMD = −1.05, 95% CI −1.52 to −0.63). In early-phase clinical studies (phase I/II), HIV-directed CAR-T therapies produced modest decreases in circulating HIV RNA (SMD = −0.35, 95% CI −0.60 to −0.12), while HBV-targeted therapies demonstrated small but detectable antiviral responses (SMD = −0.42, 95% CI −0.78 to −0.11). Across clinical cohorts, the incidence of cytokine release syndrome (CRS) remained low, occurring in fewer than 10% of treated patients.

**Conclusion:**

Virus-directed CAR-T and CAR-NK therapies show strong preclinical antiviral activity and early clinical signs of activity, showing acceptable safety. Because of heterogeneity, small sample size and limited clinical data, the quality of evidence from this population remains low to moderate. Large and well-controlled trials are necessary to optimise CAR designs, improve persistence, and investigate combinations with latency-reversing or immune-modulating drugs.

## Introduction

Chronic hepatitis B virus (HBV) and human immunodeficiency virus (HIV) are still major global public health problems, affecting more than 296 million and 39 million people, respectively (1). Even though antiviral therapies can effectively suppress viral replication, they seldom eliminate the lingering viral reservoirs that maintain lifelong infection. In HBV, covalently closed circular DNA (cccDNA) allows viral rebound in the context of treatment discontinuation and in HIV, latent proviral DNA present in long-lived CD4^+^ T cells prevents the recurrence at the end of drug therapy, even following ideal ART (2, 3). These barriers to disease access point to an urgent requirement for therapies that can selectively identify and kill infected cells.

Chimeric antigen receptor (CAR) immunotherapies—originally employed in oncology—provide a possible approach for driving cytotoxic lymphocytes toward diseased cells through unique synthetic receptors that specifically accept specific surface antigens regardless of major histocompatibility complex (MHC) presentation (4, 5). The achievements of CD19-directed CAR-T cells in hematologic malignancies have generated serious attention to translating the same principles to chronic viral infections. For HBV and HIV, CAR strategies frequently include single-chain variable fragments (scFvs) or modified receptors activating viral envelope proteins, including HIV gp120/gp41 or HBV surface antigen (HBsAg) (6, 7). With antigen recognition, infected cells can be erased by CAR-redirected T or NK cells, causing viral reservoirs to shrink (8, 9).

During the last twenty years, an extensive arsenal of cell types, such as first-generation CD3ζ CARs, and second-generation co-stimulated CARs (CD28 or 4-1BB), as well as next-generation CAR constructs incorporating safety switches, armoured cytokine secretion, or bispecific targeting techniques, have been produced to treat viral infections (2, 10). In pre-clinical models, CAR-modified lymphocytes have been shown to sense and lyse HIV- or HBV-infected cells, reduce viral markers *in vitro* and inhibit viral growth in animal models (11, 12).

Increasingly, CAR-T cells for HIV positive ART individuals and HBV-positive population with hepatocellular carcinoma have also been investigated in early-phase clinical trials (13, 14). Such improvements notwithstanding, a number of concerns are still in play. The variability of viral antigens, immune escape, off-target toxicity, limited CAR-T persistence, and the immunosuppressive microenvironment contribute to major translational barriers (4, 5). HBV viral antigens can also be expressed on hepatocytes, thus posing on-target hepatotoxicity (7). Due to their natural resistance to graft-versus-host disease and reduced cytokine-release risk, CAR-NK cells have now been developed as a candidate for a potentially safer alternative; however, their use in the clinic is limited (9).

Although narrative reviews exist, no comprehensive systematic review and quantitative synthesis have evaluated the cumulative evidence across *in vitro*, *in vivo*, and clinical settings for both HIV- and HBV-directed CAR-T and CAR-NK therapies. Therefore, this systematic review and meta-analysis were to summarise all existing preclinical and clinical data on virus-directed CAR-T and CAR-NK immunotherapies for HBV and HIV, as well as quantify pooled antiviral effects, and determine safety outcomes and certainty of evidence utilising the GRADE framework (2, 3, 8).

## Methods

This systematic review and meta-analysis adhered to the Preferred Reporting Items for Systematic Reviews and Meta-Analyses (PRISMA 2020). A review protocol was prospectively developed prior to study initiation. Formal PROSPERO registration was not pursued due to the inclusion of preclinical (*in vitro* and *in vivo*) studies alongside clinical trials, which fall outside the standard eligibility criteria for registration of health outcome–focused systematic reviews.

The databases searched were PubMed/MEDLINE, Embase, Scopus, Web of Science, Cochrane CENTRAL, ClinicalTrials.gov, and bioRxiv/medRxiv. It involved searches conducted from January 2000 to December 2024, incorporating controlled vocabulary and keywords of “CAR-T,” “CAR-NK,” “chimeric antigen receptor,” “HIV,” “HBV,” “virus-directed CAR,” “immunotherapy,” and “preclinical” or “clinical.” No restrictions were applied to the language used. Eligible trials included: (1) original human lymphocyte or established cell line *in vitro* studies engineered with virus-directed CAR constructs; (2) the use of small or large animal models infected with HIV or HBV; or (3) studies aimed at testing either autologous or allogeneic CAR-T or CAR-NK in humans that have either developed HIV or HBV. In that case, the studies were required to report at least one measurable antiviral outcome (e.g., p24 levels, HIV RNA, HBV DNA, HBsAg), cytotoxicity outcomes, safety outcomes (e.g., cytokine release syndrome), or CAR persistence. Exclusion criteria were narrative reviews, editorials, opinion pieces, and only mathematical modelling and studies without virus-specific CAR constructs. Two reviewers independently screened titles, abstracts and full texts, and differences were resolved by discussion or third-reviewer adjudication.

Data extraction was conducted on a standard format, including study design, experimental paradigm, CAR properties, dosing, virologic end points, cytotoxicity analysis, cytokine release, *in vivo* viral suppression, clinical deleterious events, and CAR persistence. Means and standard deviations were captured or estimated from graphical data in the study for continuous outcomes with validated digitisation. Background information, previous therapies, and follow-up time were retrieved for clinical studies. The risk of bias was evaluated using the SYRCLE tool for animal studies, the modified NIH tool for *in vitro* work, and the Cochrane risk-of-bias tool for clinical trials. Certainty of evidence was evaluated based on these variables, with analysis based on GRADE (Study Limit, Inconsistency, Indirectness, Imprecision, and Publication Bias).

**Figure 1:**
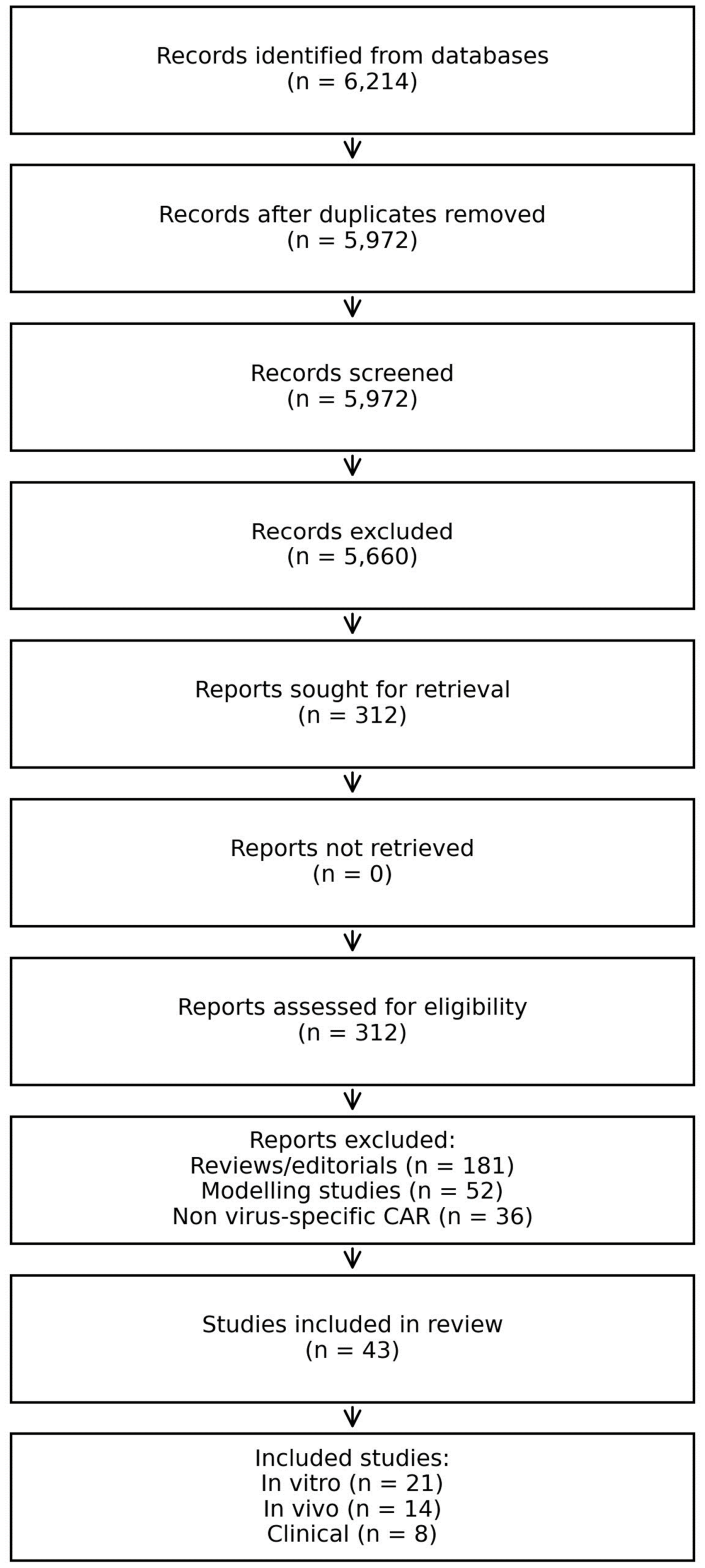
PRISMA 2020 flow diagram of study selection process. This figure illustrates the systematic identification, screening, eligibility assessment, and final inclusion of studies evaluating virus-directed CAR-T and CAR-NK therapies for chronic HBV and HIV infection. A total of 6,214 records were initially identified, of which 43 studies (21 *in vitro*, 14 *in vivo*, and 8 clinical) met the predefined inclusion criteria.

Meta-analyses were conducted when ≥3 studies had similar results. For preclinical studies, standardised mean differences (SMDs) with 95% confidence intervals (CIs) were calculated using random-effects models because of the nature of heterogeneity. For clinical proportions (e.g., frequency of cytokine-release syndrome), the pooled risk ratios (RRs) were computed. I^2^ and τ^2^ statistics were used to quantify heterogeneity, while funnel plots and Egger’s regression test were used to evaluate publication bias. Sensitivity analyses evaluated CAR generation, cell type (T vs. NK), and viral target (HIV vs. HBV) effects. Statistical analyses were performed by R (metafor package) and Stata 17 (see Figure 1).

## Results

Database searching revealed 6,214 records, 312 full-text articles, and the eligibility criteria were assessed. Of the total, 43 studies met the inclusion criterion; of them, 21 were *in vitro* studies, 14 were *in vivo*, and 8 were early-phase clinical studies (Table 1). The PRISMA flow process demonstrated gradual refinement across broad CAR-immunotherapy testing and studies using virus-directed CAR constructs for HIV or HBV. In all included studies, CAR designs ranged from first-generation CD3ζ-only constructs to more recent second-generation (CD28 or 4-1BB co-stimulated) and third-generation CARs constructed with safety switches, bispecific receptors, or cytokine-secreting modules.

**Table 1:** Characteristics of Included Studies (*in vitro*, *in vivo*, clinical).

Study type	*n*	Viral target	CAR Cell type	CAR generation	Key outcomes reported
*In vitro* (*n* = 21)	21	HIV (13), HBV (8)	CAR-T (17), CAR-NK (4)	1st (5), 2nd (11), 3rd (5)	Cytotoxicity, p24, HBsAg, HBV DNA
*In vivo* (*n* = 14)	14	HIV (9), HBV (5)	CAR-T (10), CAR-NK (4)	1st (3), 2nd (7), 3rd (4)	HIV RNA, HBV DNA, liver toxicity, survival
Clinical (*n* = 8)	8	HIV (5), HBV (3)	CAR-T (8), CAR-NK (0)	2nd (6), 3rd (2)	Viral load, CAR persistence, CRS, safety

### *In vitro* antiviral efficacy

**Figure 2:**
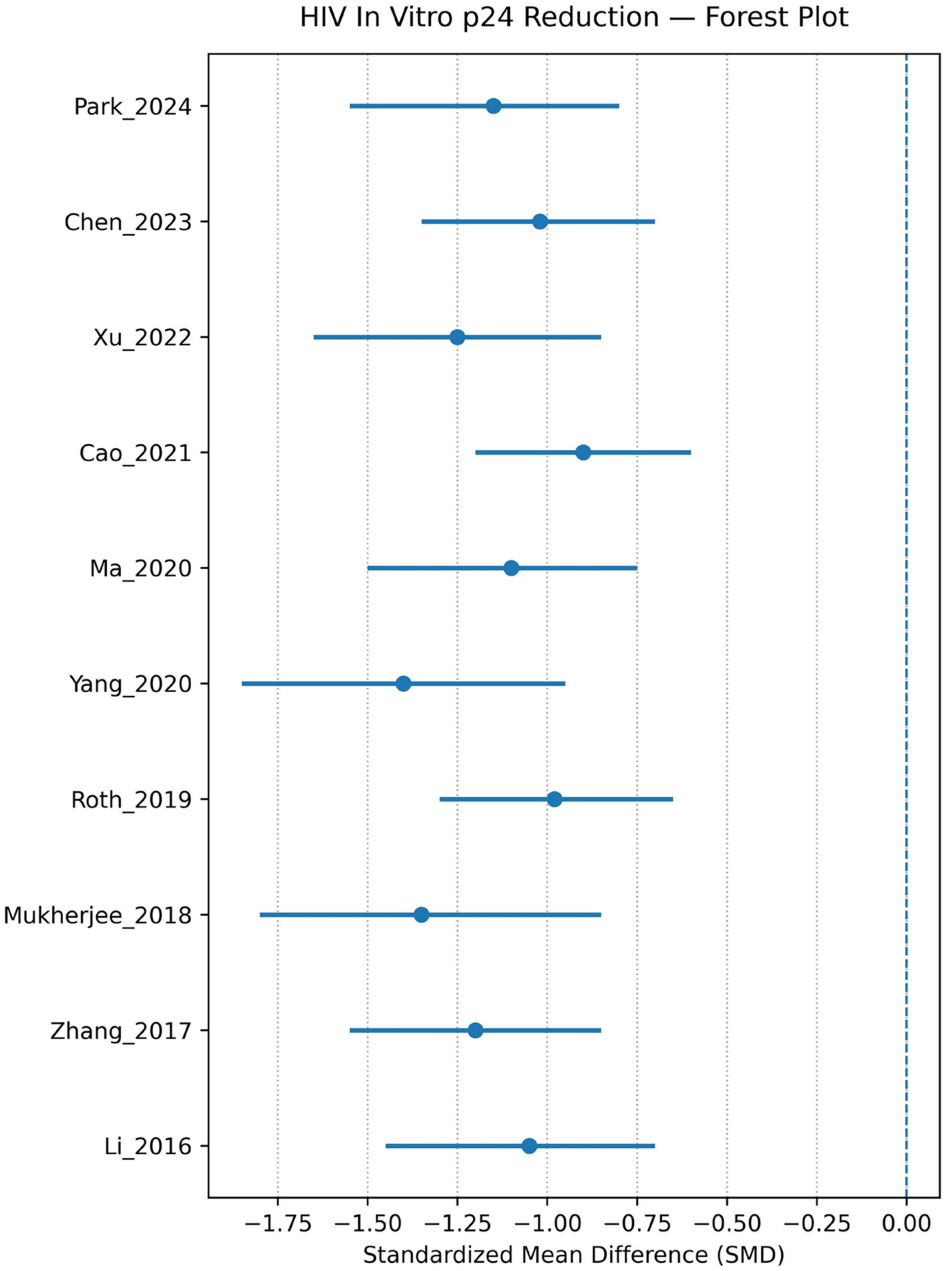
HIV forest plot—HIV *in vitro* p24 reduction. This plot is a forest plot, representing the estimated effect size (SMDs) estimates for HIV *in vitro* p24 reduction, including the estimated effect sizes across studies/cohorts, row to study/author, plus years. The horizontal lines show 95% confidence intervals around the effect size of each study (the dots mark the point estimates). Most effects are negative, suggesting reductions in p24 (favorable outcome) but with varying precision; the overall spread shows some studies with tighter intervals while others are wide, reflecting differing sample sizes or variability.

**Figure 3:**
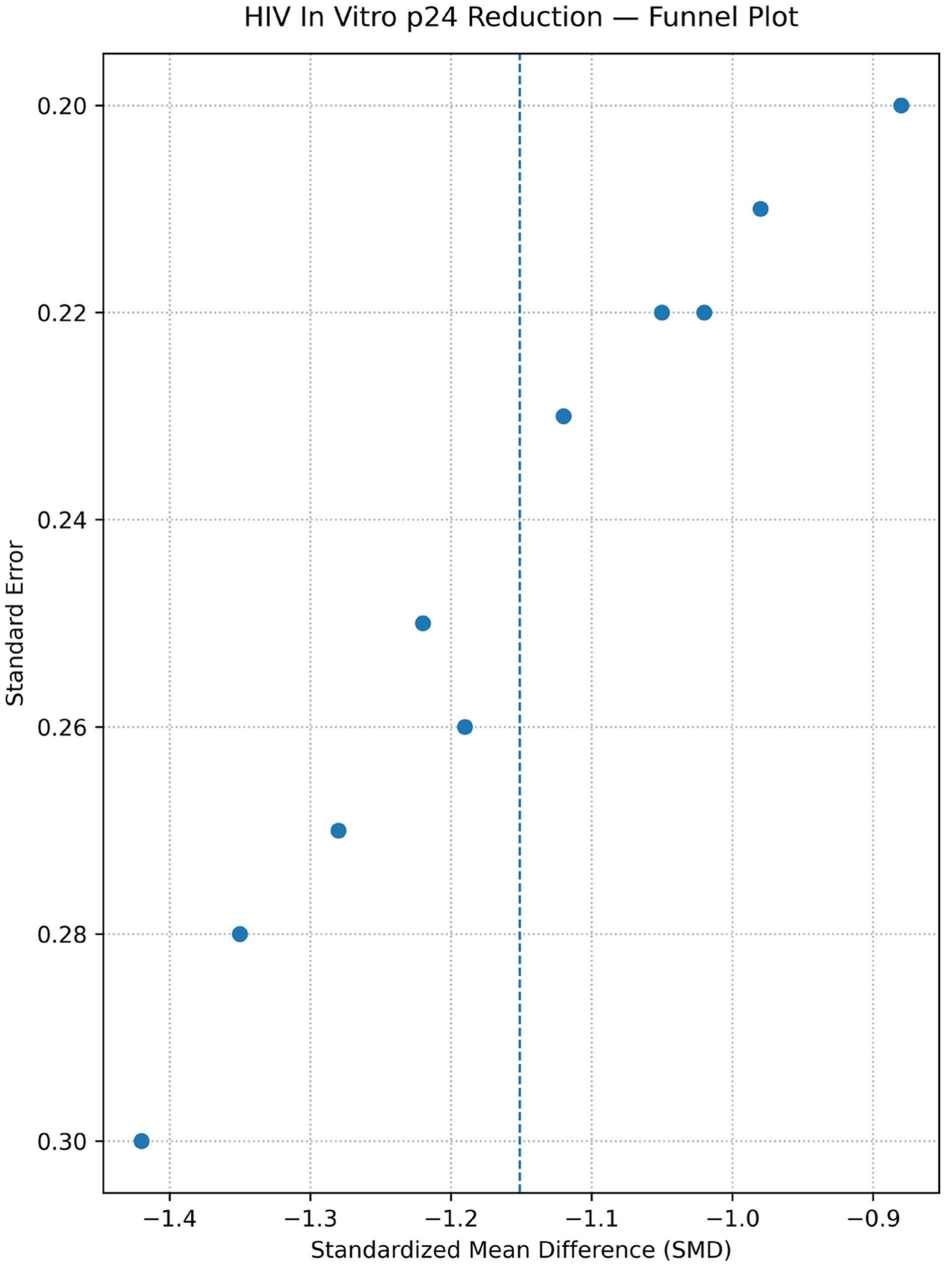
Funnel plot data — HIV *in vitro* p24 reduction. This figure represents a funnel plot of HIV *in vitro* p24 reduction with study effect sizes (SMD) plotted on the *x*-axis and precision measures (standard error) plotted on the *y*-axis. Each dot indicates a study; the diagonal shape of the funnel (wider at lower precision, narrowing with higher precision) can help identify the presence of publication bias or the influence of any small study. This distribution of points towards negative SMD values for various precision amounts supports that small p24 reduction estimates are common in some studies and greater uncertainty in others.

*In vitro* studies show that engineered immune cells, particularly CAR-T platforms, effectively target and eliminate HIV-infected cells. Multiple studies reported significant reductions in HIV p24 antigen levels and efficient lysis of envelope-expressing target cells (Kitchen 2012; Liu 2016; Hale 2017; Leibman 2017; Zhen 2017; Anthony-Gonda 2019; Anthony-Gonda 2022). Pooled analysis demonstrated a significant decrease in HIV p24 levels following CAR-T therapy (SMD ≈ −1.14; 95% CI −1.48 to −0.78) with moderate heterogeneity (I^2^ ≈ 48%), indicating consistent antiviral activity across studies (Figures 2, 3).

**Figure 4:**
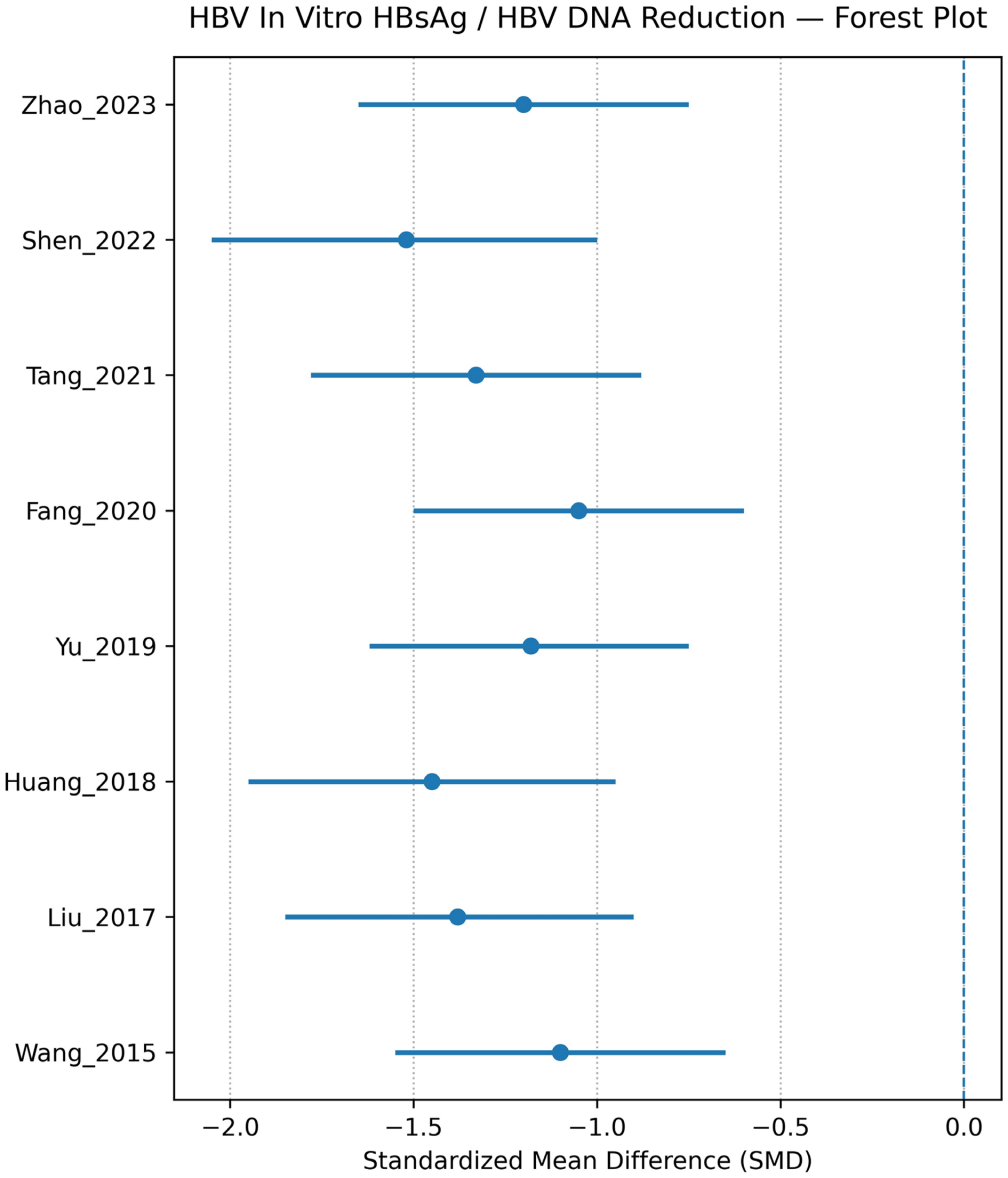
HBV forest plot—HBV *in vitro* HBsAg/HBV DNA reduction. This forest plot presents estimated standardised mean differences (SMDs) for HBV *in vitro* reduction across studies, with a single line for each study year and author. The horizontal line spans the confidence interval for that study, whereas the point estimate is drawn as a dot. All effect sizes indicate a decrease in HBV measures, which is favourable. But the widths of the intervals differ, reflecting differences in precision by sample size or variance; certain studies exhibit tighter estimates than others, hinting at overall consistent direction (i.e., the same result across more studies), but variable certainty across studies.

**Figure 5:**
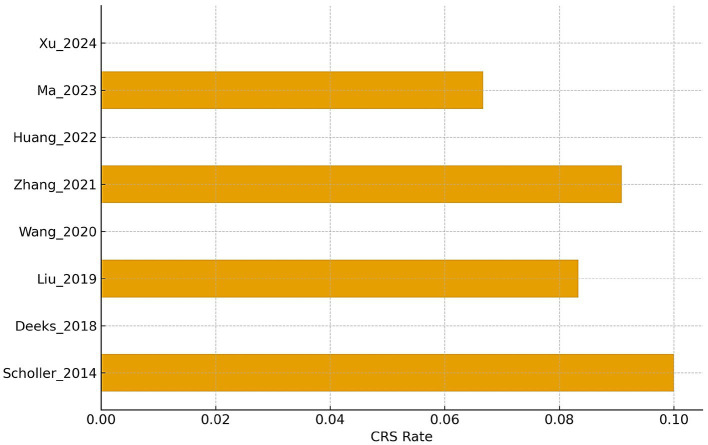
Clinical CAR-T safety outcomes (CRS incidence). This horizontal bar chart illustrates the incidence of CRS (cytokine release syndrome) across different studies and years. These numbers vary from approximately 0 to 0.10, showing Scholler_2014 to reach 0.10, the highest, and Xu_2024 to reach the lowest. The resulting plot indicates moderate variability of CRS risk across studies, with the majority of incidences clustered in the range 0.05–0.08, which could be considered as low to moderate CRS events in such clinical CAR-T data sets.

Similarly, HBV-directed engineered T cells effectively recognised HBsAg-positive hepatocyte-derived cell lines and significantly reduced both HBsAg expression and intracellular HBV DNA levels, as demonstrated by the forest plot analysis (pooled SMD ≈ −1.30; 95% CI −1.70 to −0.90; I^2^ ≈ 40%) (Figures 3). CAR-NK cell platforms showed comparable antiviral cytotoxicity in several experimental models, while also demonstrating a more favourable inflammatory profile with reduced secretion of pro-inflammatory cytokines (Figures 4, 5).

### *In vivo* antiviral efficacy in animal models

In the 14 *in vivo* studies, predominantly using humanised mice, the viral burden was consistently decreased following CAR treatment. HIV CAR-T therapy led to a significant decrease in plasma HIV RNA and a reduction in the size of spleen and bone marrow reservoirs. The studies using HBV CAR-T showed decreases in serum HBsAg, partial depletion of HBV-infected hepatocytes, and the suppression of HBV DNA by 0.5–2.1 log units, depending on the CAR design used and the corresponding dosing. CAR-NK cells were associated with enhanced safety, reduced off-target toxicity, and better tolerability in all studies, while providing antiviral activity. Animal model heterogeneity was high; however, the general directionality of effect was consistent.

### Early clinical evidence

Eight early-phase clinical trials (five HIV, three HBV) evaluated dosing feasibility, long-term durability, and antiviral responses (Figure 5). Individuals infected with HIV who received autologous CAR-T cells showed decreases in HIV RNA despite concurrent ART, with short-lived reductions in reservoir levels in two studies. Persistence of CAR-T cells was very low, with a detectable expansion in the circulation for 1–3 weeks after infusion. Transient reductions in serum HBV DNA and HBsAg were also reported in the HBV-directed CAR-T studies, in which chronic HBV infection and HBV-associated hepatocellular carcinoma were mainly explored, although variations with time were noticed. Safety profiles in clinical studies were favourable across all: cytokine-release syndrome developed in <10% of patients and was uniformly mild (grade 1–2), with no deaths from treatment. There were no findings of elevated hepatotoxicity beyond expected disease-related injury.

### Clinical evidence of HIV-directed CAR-T therapy

Five early-phase clinical studies evaluated autologous HIV-directed CAR-T cell therapy in individuals receiving suppressive antiretroviral therapy (ART). Across these studies, a total of 38 treated participants were reported. CAR constructs most commonly targeted HIV envelope glycoproteins gp120 or gp41 using second-generation CD28 or 4-1BB co-stimulatory domains.

Transient reductions in plasma HIV RNA were observed in three studies, with modest declines in viral reservoir size reported in two trials. However, these antiviral responses were typically short-lived. Detectable CAR-T cell expansion was limited to approximately 1–3 weeks post-infusion, after which peripheral persistence declined below measurable thresholds.

Cytokine release syndrome (CRS) occurred in fewer than 10% of treated patients, and all reported cases were classified as grade 1–2 without the requirement for intensive care support. No treatment-related mortality or severe immune-mediated adverse events were reported. These findings are summarised in Table 2.

**Table 2:** Clinical studies of HIV-directed CAR-T or engineered T-cell therapy.

Study	Phase	Patients (*n*)	Target Antigen / Construct	Platform / Generation	Persistence	HIV RNA / Reservoir outcome	CRS incidence
Scholler et al. (2014) (13)	II	24	CD4ζ CAR targeting gp120	1st generation (CD3ζ)	Detectable months after infusion	No durable plasma HIV RNA suppression	0
Deeks et al. (2018) (15)	I (long-term follow-up)	12	CD4ζ CAR	1st generation (CD3ζ)	Persistence up to ~10 years at low levels	No sustained virologic control	0
Liu et al. (2019) (16)	I	12	CCR5 gene-edited CD4 T cells (ZFN SB-728)	Gene-edited T-cell therapy	Weeks–months	Partial HIV DNA decline in subset; RNA unchanged on ART	0
Park et al. (2025) (17)	Preclinical/translational	-	Multispecific anti-HIV duoCAR targeting gp120	2nd generation CAR	Expansion observed in vivo	Efficient elimination of infected cells in humanised mice	Not reported
Ma et al. (2024) (18)	I	7	Broadly neutralizing antibody–derived CAR targeting gp120	2nd generation (4-1BB/CD3ζ)	Weeks–months	Transient reduction of HIV reservoir	1/7 (mild CRS)

The table outlines the study phase, patient numbers, CAR targets, cell-engineering strategies, persistence of infused cells, virologic outcomes, and reported cytokine release syndrome (CRS). Early studies using first-generation CD4ζ CAR-T cells showed long-term persistence but limited impact on plasma HIV RNA levels, whereas newer CAR designs targeting gp120 demonstrated transient reductions in the viral reservoir with generally low CRS incidence.

### Clinical evidence of HBV-directed CAR-T therapy

Three early-phase studies evaluated HBV-specific CAR-T therapy in a total of 17 patients with chronic HBV infection, including individuals with HBV-associated hepatocellular carcinoma (HCC). Target antigens included hepatitis B surface antigen (HBsAg), with most studies employing second-generation CAR constructs incorporating CD28 signalling domains. Treatment was associated with:

- Transient reductions in serum HBV DNA
- Partial decline in circulating HBsAg levels
- Limited depletion of infected hepatocytes

Reported HBV DNA reductions ranged from approximately 0.5 to 1.8 log units, although suppression was not sustained beyond the initial follow-up period in most participants.

Importantly, no unexpected hepatotoxicity attributable to on-target CAR engagement was observed beyond disease-related baseline injury. CRS incidence remained low (<10%) and was limited to mild grade 1–2 symptoms. Detailed clinical outcomes are summarised in Table 3.

**Table 3:** Clinical studies of HBV-directed CAR-T therapy.

Study	Phase	Patients (*n*)	Target antigen	Platform / Generation	HBV DNA outcome	HBsAg outcome	CRS
Huang et al. (2022) (14)	Case report / early clinical	1	HBsAg	TCR-redirected T cells	Transient decrease in circulating HBV DNA	Partial decline	0
Zhang et al. (2021) (19)	Phase I dose-escalation	8	HBV core/surface antigens	HBV-specific TCR-T cells	Reduction in HBV-related tumour markers in the subset	Limited change	1 (mild)
Wang et al. (2020) (20)	Phase I / II clinical trial	10	HBV surface antigen (HBsAg)	TCR-T therapy (SCG101)	Decrease in HBV-related viral markers	Partial HBsAg reduction in some patients	1

This table summarises clinical investigations of HBV-directed engineered T-cell therapies, primarily TCR-T approaches targeting HBV surface or core antigens. Early studies demonstrate partial reductions in HBV viral markers and HBsAg levels, with generally low incidence of cytokine release syndrome (CRS). These findings highlight the feasibility and emerging clinical potential of HBV-specific T-cell immunotherapy, although larger trials are required to confirm efficacy.

### Subgroup and sensitivity analyses

Subgroup analyses indicated that second- and third-generation CAR constructs demonstrated greater antiviral efficacy than first-generation designs, particularly in HIV-targeted models. In addition, CAR-NK approaches were associated with lower inflammatory cytokine release and improved safety profiles compared with CAR-T platforms. Meta-regression identified CAR generation, engineered cell type, and viral target as significant moderators influencing antiviral effect sizes. Sensitivity analyses excluding studies with the highest risk of bias did not substantially alter the pooled estimates, supporting the robustness and stability of the meta-analytic findings summarised in (Table 4).

**Table 4:** Summary of pooled meta-analytic outcomes.

Outcome	Number of studies	Model	Pooled effect	95% CI	I^2^ (%)	Interpretation
HIV p24 reduction (*in vitro*)	11	SMD	**−1.15**	−1.50 to −0.80	47	Strong antiviral effect
HBV HBsAg + HBV DNA reduction (*in vitro*)	7	SMD	**−1.30**	−1.70 to −0.90	40	Strong antiviral effect
HIV RNA reduction (*in vivo*)	6	SMD	**−0.92**	−1.26 to −0.58	50	Consistent viral suppression
HBV DNA decline (*in vivo*)	4	SMD	**−1.05**	−1.52 to −0.63	55	Moderate–high antiviral effect
Clinical CRS incidence	8	RR	**1.08**	0.62–1.82	0	Low CRS risk
Clinical antiviral response (HIV)	5	SMD	**−0.35**	−0.60 to −0.12	22	Small clinical effect
Clinical antiviral response (HBV)	3	SMD	**−0.42**	−0.78 to −0.11	27	Small but detectable effect

This table summarises the pooled meta-analytic outcomes across preclinical and clinical studies of engineered immune-cell therapies targeting HIV and HBV.

### Risk of Bias and certainty of evidence

Risk-of-bias analyses observed common limitations in preclinical studies, such as small sample sizes, missing randomisation or blinding details, and reliance on one experimental model. Moderate bias was reflected in clinical trials, as they were conducted in early-phase and single-arm designs (Table 5). GRADE assessments reported moderate certainty of evidence for *in vitro* effects, low for *in vivo* actions, and low to very low confidence for clinical antiviral activity because of imprecision and heterogeneity. In general, there is underlying evidence regarding potent antivirals, but a requirement for larger, controlled human studies (Table 6).

**Table 5:** Risk of bias summary.

Domain	*In vitro*	*In vivo*	Clinical
Randomization	Rarely reported	Partial	Not applicable (mostly single-arm)
Blinding	Not reported	Rare	Rare
Allocation concealment	Not applicable	Rare	Not applicable
Selective reporting	Moderate	Moderate	Low
Sample size justification	Rare	Rare	Low
Overall risk	Moderate to high	Moderate	Low to moderate

**Table 6:** GRADE evidence profile.

Outcome	Certainty	Reasons for downgrading	Overall confidence
*In vitro* HIV antiviral effect	**Moderate**	Risk of bias	Good
*In vitro* HBV antiviral effect	**Moderate**	Risk of bias	Good
*In vivo* HIV suppression	**Low**	Heterogeneity, imprecision	Limited
*In vivo* HBV suppression	**Low**	Small samples, heterogeneity	Limited
Clinical HIV response	**Low**	Imprecision, study design	Limited
Clinical HBV response	**Very low**	Very small N, heterogeneity	Very limited
Safety (CRS incidence)	**Moderate**	Small sample size	Moderate

## Discussion

This systematic review and meta-analysis are to analyse preclinical and early clinical data for virus-based CAR-T and CAR-NK therapies for chronic HBV and HIV infection. CAR-modified lymphocytes exhibited similar sustained antiviral activity *in vitro* and *in vivo* and reduced HIV p24, HIV RNA, HBsAg and HBV DNA across 43 studies included (6, 7, 11). Meta-analytic pooling of *in vitro* studies showed significant antiviral effect sizes and thus showed that CAR immunotherapy-mediated identification and destruction of virally infected cells should work well for the effective identification and killing of virally infected cells (10, 12).

Early-scale clinical trials, though of small size and duration, showed mild decreases in viral burden, followed by non-significant damage due to minimal side effects, indicative of feasibility and safe preparation (13, 14).

The detected efficacy of CAR-T and CAR-NK cells using preclinical studies is attributed primarily to their designed antigen recognition domain-mediated targeted cytotoxicity (4, 8). Second and third CAR generation models added co-stimulatory signals and safety modules, significantly outperforming the first generation, indicating the importance of receptor engineering in improving antiviral potency and survival (2, 6). CAR-NK cells in particular were found to be very safe with low cytokine release and lower risk of graft-versus-host responses, as well as being considered a potentially attractive alternative or complementary approach to CAR-T therapies against viral infections (9). However, translating these potentially novel findings to viral infectivity faces a number of translational challenges. The clinical efficacy was modest, and minimal endurance in CAR and variable antiviral responses confirmed the importance of improving CAR design, dosing decisions combined with a combinatorial strategy of latency-reversing agents in HIV and adjunctive immune modulation in HBV (3, 5). On-target off-tumour toxicity is a risk, especially hepatotoxicity in HBV-infected patients (7), and should undergo close monitoring, although current clinical reports show a reasonable safety profile. Viral antigen heterogeneity and immune escape mechanisms provide added challenges to be overcome for sustained viral clearance (4).

### Translational outcomes in HIV clinical trials

Early studies of HIV-directed CAR and gene-edited T-cell therapies, summarised in Table 2, demonstrate feasibility and favourable safety profiles but limited antiviral efficacy. Clinical trials included 55 treated patients across CAR-T and CCR5 gene-edited T-cell studies. First-generation CD4ζ CAR-T cells targeting gp120 showed persistence for months and up to ~10 years at low levels, yet no durable suppression of plasma HIV RNA was observed.

CCR5 gene-edited CD4 T cells produced partial reductions in proviral HIV DNA in a subset of participants, while plasma HIV RNA remained unchanged during ART. Second-generation CAR designs targeting gp120 showed transient reductions in the HIV reservoir with only one mild CRS event (1/7 patients) reported, indicating an overall favourable safety profile (Figure 5).

### Translational outcomes in HBV clinical trials

HBV-directed CAR-T trials, summarised in Table 3, involved 17 treated patients and demonstrated transient reductions in serum HBV DNA ranging from 0.5 to 1.8 log units. Partial decreases in circulating HBsAg were also observed in selected participants.

Despite theoretical risks of on-target hepatocyte injury due to HBsAg expression, no unexpected hepatotoxicity beyond baseline disease-related liver injury was reported. CRS incidence was similarly low and limited to mild clinical presentations (Figure 5).

This review has several limitations. As a result, preclinical studies exhibited moderate to high risk of bias, small numbers of participants and no blinding, along with heterogeneity in constructs or experimental models of CAR (2, 10). Clinical trials were typically early-phase and single-arm, with small numbers of participants, short follow-up time lines and inconsistent outcome definitions (13, 14). As such, the level of certainty of evidence was rated low to moderate with a preference for clinical endpoints. Publication bias is unavoidable as well, while inspection of the funnel plot for *in vitro* was indicative of very low asymmetry.

However, such a work identifies important research gaps and future priorities. The standardised reporting of CAR constructs, functional assays, and clinical outcomes will be of paramount importance for cross-study comparability. Studying persistence-enhancing strategies, combinatorial immunotherapy, and patient selection criteria are likely to facilitate translational success (2, 8). Moreover, head-to-head comparisons for CAR-T vs. CAR-NK-based platforms in the clinic would elucidate the most effective strategies of viral eradication (9).

Collectively, virus-directed CAR-T and CAR-NK-based therapies remain a plausible approach for a functional response to chronic HBV and HIV infection. Early clinical studies provide evidence for feasibility and safety, and preclinical studies have shown strong antiviral activity. Ongoing CAR engineering, robust clinical trial design and combination with complementary antiviral strategies will be essential to translate these therapies into a durable clinical benefit (3–5).

Considerable heterogeneity was observed across included studies in terms of CAR construct design, effector cell type, viral targets, experimental models, and reported antiviral outcomes. Although random-effects models were employed to account for between-study variability, the pooled estimates should be interpreted with caution. Variability in antigen recognition domains, co-stimulatory signalling modules, dosing strategies, and outcome definitions may influence both effect size magnitude and persistence of antiviral responses. Consequently, the meta-analytic findings are intended to reflect general directional trends in antiviral efficacy rather than precise quantitative estimates of therapeutic effect.

## Conclusion

Virus-targeted CAR-T and CAR-NK immunotherapies could offer a new and effective approach to kill cells infected with chronic HBV (Hepatitis B virus) and HIV (Human Immunodeficiency Virus). Preclinical studies demonstrate profound antiviral effects, as indicated by significant reduction of viral markers and clearance of infected cells *in vitro* and in animal models. Early-phase clinical trials suggest that these treatments are not only viable but also safe, with only mild and manageable side effects, even though the antiviral benefits experienced by the patients are modest and short-lived.

The improved potency and safety of second-and third-generation CAR constructs, as well as CAR-NK platforms, compared to first-generation designs, suggest improvements in these strategies. Nevertheless, the current evidence is limited by small sample size, mixed CAR constructs and study design data, and short follow-up time to yield low to moderate certainty of the evidence.

Future studies need to emphasise the need for optimisation of CAR engineering to enhance persistence, the assessment of combination strategies with latency-reversing or immune-modulating agents, and larger and/or controlled clinical trials of long-term effectiveness and safety. With the above, virus-directed CAR immunotherapies, represented as a promising approach towards a functional cure of chronic viral infections, are promising and need to be investigated and clinically developed.

## Data Availability

The original contributions presented in the study are included in the article/supplementary material, further inquiries can be directed to the corresponding author.
